# Pretreatment Glasgow Prognostic Score Correlated with Serum Histidine Level and Three-Year Mortality of Patients with Locally Advanced Head and Neck Squamous Cell Carcinoma and Optimal Performance Status

**DOI:** 10.3390/nu14173475

**Published:** 2022-08-24

**Authors:** Kun-Yun Yeh, Chao-Hung Wang, Hang Huong Ling, Chia-Lin Peng, Zih-Syuan Chen, Simon Hsia

**Affiliations:** 1 Division of Hemato-Oncology, Department of Internal Medicine, College of Medicine, Chang Gung Memorial Hospital, Keelung 20401, Taiwan; 2 College of Medicine, Chang Gung University, Taoyuan 333007, Taiwan; 3 Heart Failure Research Center, Division of Cardiology, Department of Internal Medicine, Chang Gung Memorial Hospital, Keelung 20401, Taiwan; 4 Taiwan Nutraceutical Association, Taipei 104483, Taiwan

**Keywords:** head and neck cancer, metabolites, Glasgow prognostic score, histidine, inflammation, performance status

## Abstract

Few prospective cohort trials have investigted the effect of pretreatment nutritional and inflammatory status on the clinical outcome of patients with cancer and optimal performance status and assessed the interplay between nutrition, inflammation, body composition, and circulating metabolites before treatment. Here, 50 patients with locally advanced head and neck squamous cell carcinoma (LAHNSCC) and Eastern Cooperative Oncology Group performance status (ECOG PS) ≤ 2 were prospectively recruited along with 43 healthy participants. Before concurrent chemoradiotherapy, compared with healthy controls, the cancer group showed lower levels of histidine, leucine, and phenylalanine and had low values in anthropometric and body composition measurements; however, the group displayed higher ornithine levels, more malnutrition, and severe inflammation. Pretreatment advanced Glasgow prognostic score (1 and 2) status was the sole prognostic factor for 3-year mortality rate and was associated with age and serum histidine levels in patients with cancer. Thus, even at the same tumor stage and ECOG PS, patients with LAHNSCC, poor nutrition, and high inflammation severity at baseline may have inferior survival outcomes than those with adequate nutrition and low inflammation severity. Assessment of pretreatment nutritional and inflammatory status should be included in the enrollment criteria in future studies.

## 1. Introduction

Most patients with head and neck cancer (HNC) present with locally advanced disease [1]. High prevalence of inadequate nutritional status and severe inflammatory response have been observed in patients with locally advanced head and neck squamous cell carcinoma (LAHNSCC) at the time of diagnosis [2,3,4,5]. A malnourished and hyperinflammatory condition imposes a significant negative impact on the treatment regimen tolerability, quality of life, and survival outcomes of patients with LAHNSCC [3,6,7,8]. However, in oncologists’ routine clinical practice, the assessment of nutritional and inflammatory status is not regularly made and is also not routinely required during prospective cancer studies. This is mainly due to the lack of clinicians’ awareness and scarcity of convincing randomized control trials [9,10,11], substantially weakening the importance of a clinical nutritional assessment in parallel with conventional cancer stage examination and treatment [12]. Furthermore, it is possible that patients with LAHNSCC who have been suffering from poor nutrition and severe inflammation show inferior treatment outcomes than that of those with adequate nutrition and no inflammation even in the equivalent and optimal medical performance status. Hence, proper assessment of nutritional and inflammatory status before treatment should be conducted, and high-quality prospective trials are required to improve oncologists’ awareness of such an issue. For patients with LAHNSCC and optimal performance status, no appropriate prospective trial regarding the interplay between pretreatment nutritional and inflammatory status and survival outcome prediction has been reported.

Certain clinicopathologic variables, nutrition-inflammation biomarkers (NIBs), and body composition parameters can assess pretreatment malnourished status, reflect the severity of systemic inflammation, and predict the outcomes of patients with HNC [13,14,15]. These include old age [14], comorbidity [14,15], poor performance status [13,14], low body mass index (BMI) and body weight (BW) [13,14,16], anemia [14,15], low total lymphocyte count [13], low prognostic nutritional index (PNI) [17], high levels of C-reactive protein (CRP), neutrophil-to-lymphocyte ratio (NLR), platelet-to-lymphocyte ratio (PLR) [18,19,20], and total muscle and fat mass [19,21]. In particular, the Glasgow prognostic score (GPS), an inflammation-based model that combines the levels of serum albumin and CRP, has demonstrated significance for predicting outcomes in patients with HNC under different clinical circumstances [22,23,24,25,26]. Patients with HNC or oropharyngeal squamous cell carcinoma who expressed a low pretreatment modified Glasgow prognostic score (mGPS) showed better survival outcomes than those who expressed a high mGPS [23,25]. The improved GPS subgroup of patients with LAHNSCC after concurrent chemoradiotherapy (CCRT) showed a tendency toward superior overall and recurrence-free survivals [22]. GPS status after nivolumab treatment was also identified as an independent prognostic factor for the overall survival of patients with recurrent/metastatic HNC [24]. The correlation between pretreatment GPS or mGPS status and overall survival has also been reported in patients with recurrent/metastatic HNC receiving nivolumab therapy [26,27]. However, most of these studies were retrospectively conducted among heterogeneous patient populations with varied tumor stages, treatment protocols, and data collection periods. Furthermore, a comprehensive analysis which includes all the possible confounding covariates among the published reports is lacking. Although poor performance status showed an independent contribution to prognostic outcomes as well as NIBs in some studies [24,26,27], the effect of clinicopathologic factors, NIBs, and body composition on the survival outcome of patients with HNC who have good performance status remains uncertain. Therefore, these results should be interpreted with caution before clinical application.

Metabolomics offers a powerful tool to assess the biochemical changes in cells, tissues, and body fluids, helps to understand complicated disease processes, and is applied to patients with HNC [28,29,30,31]. A comprehensive review showed different metabolite profiles between healthy participants with no cancer and patients with HNC using various sample types and metabolomics platforms [28]. Although metabolomics improves our knowledge of the HNC pathogenesis, its clinical application in daily practice is limited, probably due to the complex interpretation of multi-metabolite measurements, inherent inconsistency of metabolite profiles from different tissue samples, limited availability of technology platforms, and equivocal data from heterogeneous enrollment of study patients with mixed tumor stages [29,30,31,32]. Recently, a simple and easy-to-read metabolite panel was developed using ultra-performance liquid chromatography (UPLC) to measure the serum levels of four amino acids (histidine, leucine, ornithine, and phenylalanine; HLOP) [28,33,34]. The HLOP panel, corresponding to the muscle synthesis and breakdown, nutritional status, and nitrogen waste via the urea cycle, has shown a prognostic influence in patients with severe infection [35], congestive heart failure [33,34], chronic kidney disease [36], and chronic obstructive pulmonary disease [37]. However, it remains unknown whether the HLOP profile differs between patients with HNC and healthy adults. Furthermore, the clinical implications of pretreatment levels of the HLOP-based panel in patients with HNC have not yet been studied. The interactions among metabolomics, clinicopathological variables, malnutrition status, inflammation severity, body composition parameters, and clinical outcomes also require further investigation.

To solve the confounding issues of retrospective study designs, mixed tumor stages, varied performance status, and diverse treatment protocols, a prospective, observational study was required. We prospectively enrolled a homogenous group of patients with LAHNSCC (stage III, IVA, or IVB) who had an optimal performance status, i.e., Eastern Cooperative Oncology Group performance status (ECOG PS) ≤ 2 before CCRT, and subsequently received standard CCRT (RT at a fraction daily for 5 days per week over 6–8 weeks concurrently with weekly cisplatin infusion chemotherapy). In this study, we evaluated the differences between the patients with cancer and optimal performance status and healthy controls with regard to anthropometric parameters, NIBs, and serum HLOP profiles. By simultaneously analyzing all covariates, including clinicopathological variables, anthropometric data and NIBs, body composition measurements, and the HLOP panel, we aimed to identify prognostic factors contributing to the 3-year mortality rate of patients with LAHNSCC receiving CCRT and independent factors associated with the pretreatment of these prognostic factors in patients with cancer.

## 2. Materials and Methods

### 2.1. Patient Recruitment

Eligible patients with histologically proven LAHNSCC originating in the oral cavity, oropharynx, hypopharynx, and larynx were prospectively recruited between January 2018 and July 2019. According to the 8th edition of the American Joint Committee on Cancer (AJCC) staging system and criteria of inclusion and exclusion, the HNC committee of our institute confirmed LAHNSCC and tumor stage, including stages III (T1-2, N1, or T3, N0-1), IVA (T4a, N0-1, or T1-4a, N2), and IVB (any T, N3, or T4b, any N). The inclusion criteria were age ≤75 years, optimal performance status defined as an ECOG PS score ≤2, sufficient hematopoietic or organ function, and no expression in the human papilloma virus test in tumor specimens. The exclusion criteria were as follows: (1) systemic illnesses such as enduring infections, uncontrolled diabetes mellitus, end-stage renal disease, severe chronic obstructive pulmonary disease, decompensated liver cirrhosis with intractable ascites or hepatic encephalopathy, heart failure with New York Heart Association classification IV, major gastrointestinal disorders, or autoimmune diseases and (2) consumption of medications, such as steroids or megestrol acetate, that probably interfere with metabolism or BW.

Forty-three control participants matched with the LAHNSCC population with respect to age and sex were included in this study. They had to fulfill the following requirements: (1) no active or previous malignancies, (2) no reported exposure to cigarette smoking, alcohol, or betel nut, and (3) no medications for hypertension, diabetes, dyslipidemia, endocrine illness, coronary artery disease, pulmonary disorders, or autoimmune diseases.

The median follow-up time and interquartile range for cancer patients was 27.6 months and 10.0 months, respectively. The follow-up time was defined as the period from diagnosis to the day of the last visit or death due to any cause. At the time of writing this manuscript, fourteen cancer patients have died, while all 43 control participants remain alive and have regular outpatient clinic visits.

Patients received either postoperative adjuvant CCRT for oral cavity cancer or curative-intent primary CCRT for unresectable cancers of the oropharynx, hypopharynx, and larynx. Intensity-modulated radiotherapy at a dose of 60–72 Gy in 30–36 fractions, a fraction daily for 5 days per week over a 6–8-week period, with concurrent chemotherapy with weekly cisplatin (40 mg/m^2^) was administered.

This study was approved by the Institutional Review Board (IRB) of the Chang Gung Memorial Hospital (CGMH), Taiwan (approval numbers: 101-4047B and 201700158B0), and was conducted in compliance with the Good Clinical Practice Guidelines and Declaration of Helsinki. Written informed consent was obtained from the control participants and patients with cancer upon enrollment.

### 2.2. Clinicopathological Data

Clinicopathological data, including age, sex, body height (BH), BW, ECOG PS, comorbid illness, tumor sites, tumor stage, histologic differentiation grade, status of tumor size (T), regional lymph node involvement (N), presence of tracheostomy, and exposure records of cigarette smoking, alcohol, and betel nut, were collected. Smokers were defined as current cigarette smokers or those who had been previously exposed to cigarette smoking. Alcohol drinkers were defined as those who consumed alcohol more than 4 times per week. Betel quid users were defined as patients who had consumed betel nuts during the previous year. The severity of comorbidity was assessed using the head and neck Charlson comorbidity index (HN-CCI) [38]. BMI (kg/m^2^) was determined as the BW (in kilograms) divided by the square of the BH (in meters).

### 2.3. Biochemical Data and Blood NIBs

Blood samples were collected after overnight fasting within one week before CCRT. Biochemical data and NIBs, including hemoglobin (Hb, g/dL), white blood cell count (WBC, 103/mm^3^), platelet count (103/mm^3^), albumin (g/dL), prealbumin (g/dL), transferrin (g/dL), creatinine (mg/dL), alanine transaminase (ALT, U/L), total bilirubin (mg/dL), uric acid (mg/dL), fasting glucose (mg/dL), total cholesterol (mg/dL), triglycerides (mg/dL), and CRP (mg/dL) were measured using an auto-analyzer (Beckman, CA, USA) at the CGMH central laboratory in Keelung, Taiwan.

The estimated glomerular filtration rate (eGFR, mL/min/1.73 m^2^) was calculated using the abbreviated modification of diet in renal disease study equation, corrected to a body surface area of 1.73 m^2^ [39]. Total lymphocyte count (TLC), total neutrophil count (TNC), total monocyte count (TMC), total eosinophil count (Teso), and total basophil count (Tbaso) were calculated as WBC counts (/mm^3^) × the percentages of lymphocytes, neutrophils, monocytes, eosinophils, and basophils in the blood, respectively. The NLR was calculated as the ratio of absolute neutrophil count to lymphocyte count, while PLR was calculated as the ratio of platelet count to lymphocyte count. Further, PNI = 10 × serum albumin (g/dL) + 0.005 × TLC (/mm^3^) [17]. The percentage of BW change was calculated using the following formula: [(current weight in kg-previous weight in kg)/previous weight in kg] × 100, where previous weight was defined as the BW measured in the six months preceding diagnosis. GPS was defined based on the presence of hypoalbuminemia (<3.5 g/dL) and elevated CRP (>10 mg/L) as follows: if both were abnormal, the score was 2; if either was abnormal, the score was 1; if neither was abnormal, the score was 0 [40].

The malnutrition status was determined using BW loss (BWL) > 5.0%, BMI < 18.5 kg/m^2^, albumin < 3.5 g/dL, TLC < 1.5 × 10^3^ cells/mm^3^, or the patient-generated subjective global assessment (PG-SGA). PG-SGA scores were in a range between 0 and 3, and scores of 0–3, 4–8, and ≥9 indicated no, moderately, and severely malnourished status, respectively [41,42,43].

### 2.4. Body Composition Measurements

Following the guidelines set by the International Society for Clinical Densitometry to accurately place each participant, we obtained body composition parameters, including the lean body mass (LBM), total fat mass (TFM), and appendicular skeletal mass (ASM, arm and leg), using dual-energy fan-beam X-ray absorptiometry (Lunar iDXA, GE Medical System, Madison, WI, USA) within one week before CCRT [44]. According to the BMI and body size, the scanner software selected the scan mode (standard, thin, or thick). Scans were analyzed using enCORE software, version 15 (San Jose, CA, USA).

### 2.5. Ultra-Performance Liquid Chromatography (UPLC)-Based Measurement

Blood levels of histidine, leucine, ornithine, and phenylalanine were measured as previously described [34,37]. Briefly, EDTA-treated plasma samples were harvested within one week before CCRT and within three days after CCRT. They were stored at −80 °C until assayed. Plasma samples (100 μL) were precipitated by adding an equal volume of 10.0% sulfosalicylic acid containing 200 μM norvaline (an internal standard). Derivatization was initiated by adding 10 mM AQC in acetonitrile after protein precipitation and centrifugation at 12,000× *g* for 10 min at room temperature. Eluent A (20 mM ammonium formate/1.0% acetonitrile) was added to the mixture after 10 min of incubation, and the amino acids were examined using the ACQUITY UPLC System [45,46], which consisted of a binary solvent manager, sample manager, and tunable UV detector. The system was controlled, and data were collected using Empower™ 2 software (Waters Corporation, Milford, MA, USA). Separations were conducted on a 2.1 × 100 mm ACQUITY BEH C18 column at a flow rate of 0.70 mL/min. For histidine, ornithine, leucine, and phenylalanine, the average intra-assay coefficients of variation were 4.3, 4.6, 4.5, and 4.6%, respectively, while the total coefficients of variation were 3.1, 3.6, 4.1, and 3.7%, respectively. Further, the detection limits for histidine, ornithine, leucine, and phenylalanine were 0.5 μM, 2.0 μM, 0.9 μM, and 3.3 μM, respectively. The linear range for these four amino acids was 25–500 μM.

### 2.6. Statistical Analysis

SPSS (version 22.0; SPSS Inc., Chicago, IL, USA) was used for the statistical analyses. Based on a power of 80%, α error of 0.05, and the annual number of patients with LAHNSCC receiving CCRT at our institute, the calculated minimum sample size was 42. The primary endpoint of this prospective study was the correlation between NIBs and all variables, while the secondary endpoint was 3-year mortality rate. All continuous variables were examined for normality before analysis. Independent *t*-tests, analysis of variance (ANOVA) with Bonferroni adjustments or nonparametric Mann–Whitney tests, and the Kruskal–Wallis H test were used for continuous variables, where appropriate. The chi-square test was used for categorical variables. Variables showing statistical significance (*p* < 0.05) in the univariate logistic regression analysis were employed during the multivariate logistic regression analysis to identify the independent variables associated with 3-year mortality rate and pretreatment advanced GPS in patients with LAHNSCC. Three-year mortality rate was defined as the proportion of patients who died within 1095 days of the start of treatment, which was used as the reference date due to variations in the time for stage workups. All differences with a two-tailed *p*-value < 0.05 were considered statistically significant.

Correlation matrices visualizing correlations among metabolites, biochemical and anthropometric factors, NIBs, and DXA-derived parameters were obtained using the Pearson correlation coefficient between each pair of variables and were constructed using Statgraphics Centurion version 19 (Statgraphics Technologies, Inc. The Plains, VA, USA).

## 3. Results

### 3.1. Comparison between Patients with LAHNSCC and Control Participants

Table 1 shows the clinical features of 50 patients with LAHNSCC and 43 control participants. No differences were observed in age, sex, BH, WBC, TNC, Teso, Tbaso, triglyceride concentration, hepatorenal function, NLR, or PNI between the two groups. Compared to the control group, the LAHNSCC group had significantly more comorbid illness; exposure to smoking, alcohol, and betel quid; and lower values of BW, BMI, Hb, TLC, albumin, uric acid, and cholesterol but higher levels of fasting sugar, platelet count, TMC, CRP, and PLR. Patients with LAHNSCC showed significantly higher malnutrition rates compared to the control group participants according to the different criteria (BWL > 5.0%: 26.0% vs. 2.3%, *p* = 0.001; BMI < 18.5 kg/m^2^: 18.0% vs. 0.0%, *p* = 0.003; TLC < 1.5 × 10^3^ cells/mm^3^: 34.0% vs. 14.0%, *p* < 0.001; albumin < 3.5 g/dL: 14.0% vs. 0.0%, *p* < 0.001), advanced GPS (GPS 1 + 2: 34.0% vs. 2.3%, *p* = 0.001), and displayed a more severe inflammatory status (CRP > 5 mg/L: 34.0% vs. 2.3%, *p* = 0.001). Further, patients with LAHNSCC showed lower pretreatment blood levels of histidine, leucine, and phenylalanine but higher levels of ornithine than the control participants (Table 1). Finally, we performed a PG-SGA for both healthy controls and patients with LAHNSCC. All participants in the control group reported “unchanged”, while 94% of cancer patients reported “less than usual” in the status of food intake during the month before treatment.

### 3.2. Characteristics of Patients with LAHNSCC before CCRT

Of the 50 patients with LAHNSCC, 28 with oral cavity cancer received postoperative adjuvant CCRT, while 22 with non-oral cavity cancer received primary CCRT. The patients were predominantly male (96.0%), with an average age of 54.9 years. The most common tumor site was the oral cavity (56.0%), followed by the hypopharynx (22.0%) and oropharynx (18.0%). Over 90 percent of the patients had non-metastatic TNM stage IV (IVA + IVB: 92.0%) and had an ECOG PS of ≤ 1 (92.0%). Most patients presented with advanced tumor size (T3 + T4: 70.0%), extended regional lymph node invasion (N2 + N3: 68.0%), good histologic differentiation (well + moderate: 86.0%), and exposure to cigarette smoke (84.0%), alcohol (72.0%), and betel quid (62.0%). Sixty percent of patients had at least one comorbid illness. Further, 40.0% patients underwent tracheostomy before CCRT (Table 2).

The average BMI was 22.9 kg/m^2^. Pretreatment malnutrition rates were assessed using different malnutrition criteria: PG-SGA-defined malnourished status (98.0%), BWL > 5.0% (26.0%), BMI < 18.5 kg/m^2^ (18.0%), albumin < 3.5 g/dL (18.0%), and TLC < 1.5 × 10^3^ cells/mm^3^ (34.0%). The patients with cancer and optimal ECOG PS developed varying degrees of malnutrition and inflammation severity before the start of CCRT (Table 1 and Table 2). A 3-year mortality rate of 28.0% was observed (Table 2).

### 3.3. Correlation among the Pretreatment Levels of Biochemical and Anthropometric Variables, NIBs, DXA-Related Measurements, and Serum Metabolites in Patients with LAHNSCC

**Figure 1:**
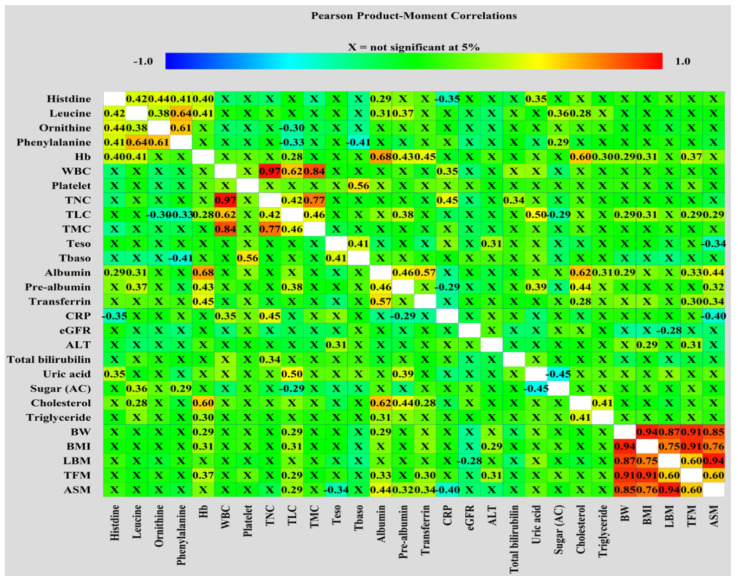
Correlation matrices used to visualize the correlations of the pretreatment levels of metabolites, biochemical and anthropometric factors, NIBs, and DXA-derived parameters were obtained using Pearson correlation coefficient.

Figure 1 shows the associations among pretreatment levels of biochemical and anthropometric variables, NIBs, DXA-related measurements, and serum metabolites. The four amino acid metabolites were positively correlated with each other. Histidine levels were positively correlated with the levels of albumin and uric acid but negatively correlated with the CRP levels. Leucine showed positive correlation with albumin, prealbumin, fasting sugar, and cholesterol levels. Ornithine and phenylalanine levels negatively correlated with TLC. Hb levels were positively correlated with histidine, leucine, albumin, prealbumin, transferrin, cholesterol, TG, BW, and BMI. Albumin positively correlated with prealbumin, transferrin, cholesterol, TG, and BW. All pretreatment values of BW, BMI, and DXA-associated measurements were positively correlated with each other. The TFM was positively correlated with Hb, TLC, albumin, transferrin, and ALT levels. ASM was positively correlated with albumin, prealbumin, transferrin, and TLC but negatively correlated with CRP levels. This correlation analysis suggests an intricate and tight relationship between the pretreatment levels of various blood-related variables and anthropometric and body composition parameters.

### 3.4. Pretreatment GPS Independently Correlated with 3-Year Mortality Rate in Patients with LAHNSCC

We further stratified patients according to their GPS status. Two-thirds of patients had a GPS of 0. We found no statistical differences in the following variables, irrespective of GPS status: sex, tumor sites, status of T and N, smoking, exposure to cigarette smoking and alcohol, the presence of tracheostomy, PG-SGA, pretreatment hepatorenal function, pretreatment WBC count, platelet count, TLC, TNC, TMC, Teso, Tbaso, uric acid, TG, BW, BMI, LBM, TFM, leucine, ornithine, and phenylalanine. Nonetheless, a higher percentage of patients with a GPS of 1 or 2 were aged ≥ 65 years and had an ECOG PS of 2; further, patients with a GPS of 1 or 2 expressed lower pretreatment levels of Hb, albumin, prealbumin, transferrin, PNI, ASM, and histidine but higher levels of NLR and CRP than those with a GPS of 0 (Table 2). Patients with an advanced GPS (score of 1 + 2) had a higher 3-year mortality rate than that of those with a low GPS (score of 0) (52.9% vs. 15.2%, *p* = 0.005).

We further investigated the prognostic factors associated with the 3-year mortality rate of patients with LAHNSCC and found that age ≥ 65 years and pretreatment levels of Hb, TNC, transferrin, cholesterol, CRP, NLP, and GPS status were the significant factors in the univariate analysis. However, only advanced GPS status independently contributed to the 3-year mortality rate in multivariate analysis (Table 3).

### 3.5. Pretreatment GPS Correlated with Age and Histidine Levels in Patients with LAHNSCC Undergoing CCRT

To further determine the factors associated with advanced GPS, we performed multivariate analysis after adjustment for all the covariates, including clinicopathologic variables, biochemical and anthropometric data, blood NIBs, body composition parameters, and levels of the four individual amino acid metabolites. Only two variables, namely, age and pretreatment histidine levels, were independently correlated with the advanced GPS of patients with LAHNSCC (Table 4).

## 4. Discussion

At the time of diagnosis, 30–50% of patients with LAHNSCC have associated with malnutrition and hyperinflammation, which consequently accounts for up to 20% of cancer-related deaths [3,47]. Lonbro et al. found that patients with HNC had significantly lower values of BW, BMI, and lean body mass than those of healthy controls [48]. Ghadjar et al. showed that 32% of patients with HNC presented with BWL > 5% before treatment, which was associated with treatment failure and inferior survival outcomes [49]. The development of malnutrition and hyperinflammation is ascribed to mechanical intake difficulty and increased catabolism due to systemic inflammation mediators, both of which are caused by cancer itself, comorbid illnesses, and excess exposure to substances or immune cells in the tumor microenvironment and peripheral blood [8,50,51,52,53,54,55,56]. Takenaka et al. reported that tumor size and comorbidity independently affected pretreatment BMI in patients with HNC [56]. Cigarette smoke constituents can directly affect the skeletal muscle tissue or indirectly stimulate the production of certain pro-inflammatory cytokines (tumor necrosis factor (TNF)-α and interleukin (IL)-6) from the lung, activated leukocytes, bone marrow, and muscle tissue, which, consequently, enhance muscle proteolysis and inhibit muscle protein synthesis, leading to skeletal muscle loss [51]. Loss of skeletal muscle mass mainly accounts for BWL in patients with HNC presenting with cachexia syndrome [57]. Alcohol can induce muscle loss and dysfunction via disruption of both anabolic and catabolic pathways of muscle mass maintenance by increasing oxidative and pro-inflammatory stress in skeletal muscle or by directly inhibiting the regenerative capacity of muscle progenitor cells [55]. In Taiwan, approximately 85% of patients with HNC have betel quid chewing habits, which independently contribute to the risk of HNC [58]. More than 50% of these patients who are betel quid users are alcohol and cigarette consumers [58]. Betel quid is formed by areca nuts usually wrapped with piper betel leaves or inflorescences to improve the chewing flavor [58]. Areca nut extracts contain several alkaloids, of which arecoline is the most abundant component, which increases reactive oxygen species, induces inflammation, and actively participates in cancer development [58]. In patients with HNC, several pro-inflammatory mediators have been reported, which are released from the cancer and immune cells into circulation and induce chronic, debilitating symptoms and sarcopenia [53]. However, these results should be further verified before clinical use because these studies were preclinical, had retrospective design, and enrolled heterogeneous study populations with varied therapeutic modes and data collection timing. The salient features of the current study are its prospective design and homogenous study population with standard data collection timelines and treatment protocols. Our results partially support the observations of previous reports on the effect of malnutrition and hyperinflammation on survival outcomes [48,49,56]. On the other hand, we also compared healthy adult controls and found that, although more than 90% of patients had an ECOG PS ≤1 before CCRT, they still had significant reductions in BW, BMI, serum levels of essential amino acids (histidine, leucine, and phenylalanine), were more likely to develop malnutrition status, and presented with higher inflammation severity (indicated by advanced GPS and elevated CRP levels). A greater proportion of patients with LAHNSCC had BWL > 5%, suffered from comorbid illness, and experienced substance exposure, including cigarette smoking, alcohol consumption, and betel quid use. Furthermore, patients with advanced GPS before CCRT had a higher 3-year mortality rate. This essential finding highlights the fact that a certain proportion of patients with LAHNSCC with an optimal ECOG PS are already at malnutrition and hyperinflammation status before CCRT and may experience a survival disadvantage, showing a more aggressive clinical course at baseline. Unfortunately, the status of nutrition and inflammation in patients with cancer is seldom recognized as one of the inclusion criteria in any clinical trials.

Accumulative evidence has focused on the establishment of easy and practical indicators to help healthcare professionals to identify malnourished and inflammatory status early. Several surrogate indicators of nutrition and inflammation have been reported as independent prognostic factors in patients with HNC [18]. However, certain limitations restrict their application in evaluating the association between malnutrition status and prognosis. First, separate anthropometric (BWL, BMI), biochemical, nutritional (albumin, pre-albumin, transferrin, Hb, and TLC), and inflammatory indicators (CRP, NLR, and PLR) are widely used as different arbitrary cutoffs that, consequently, produce inconsistent malnutrition rates and inflammation severity among studies. They are also interrelated since malnutrition could be a consequence of hyperinflammation, metabolic derangement, and immune system perturbations due to cancer itself and the patient’s own clinicopathological factors. Additionally, aggregate parameters (PG-SGA, integrating BWL-BMI grading) may offset their values in prognosis prediction because of either data recall bias or insufficient analysis of specific cancer stages [4,59]. Furthermore, although body composition parameters generated from DXA or computerized tomography provide a close understanding of muscle, fat, and other tissues that could correspond to nutrition and inflammation status and correlated the prognosis of patients with LAHNSCC receiving CCRT [19,21], cost and inconvenience limit their clinical application. In this context, GPS offers two advantages in clinical practice: ease of use with low cost and simultaneous reflection of the systematic nutritional and inflammatory status of patients with cancer [60]. In partial accordance with previous reports [22,23,24,25,26], our data found that patients with cancer and advanced GPS status were older, showed more betel quid use, and had decreased Hb levels, nutritional index (albumin, prealbumin, transferrin, and PNI), muscle mass, and histidine levels but higher levels of inflammatory markers (NLR and CRP), indicating a correlation between GPS, nutrition, and inflammation. Using the multivariate analysis that included all the possible confounding covariates, we further confirmed that advanced GPS outperformed other variables and was the sole prognostic factor for the 3-year mortality rate of patients with LAHNSCC (Table 3). These observations again strengthen that nutrition and inflammation status before treatment affects survival outcomes.

Circulating amino acid profiles are regarded as systemic biomarkers of nutritional and inflammatory status, corresponding to food intake and absorption, disease severity, comorbid illness, and tissue synthesis and breakdown [61,62]. In the current study, the levels of amino acids (HLOP panel) in patients with cancer before CCRT differed significantly from those in the control group (Table 1). In particular, histidine levels were negatively associated with GPS status, which independently predicted the 3-year mortality rate in the patients with cancer (Table 3 and Table 4). These results verified the prognostic relevance of the HLOP panel, as demonstrated in different, previously published clinical situations [33,34,35,36,37,61,62]. It is, thus, evident that the pretreatment HLOP panel in patients with LAHNSCC can represent a miniature set of circulating amino acids, showing changes in levels in response to nutrition and inflammation status.

**Figure 2:**
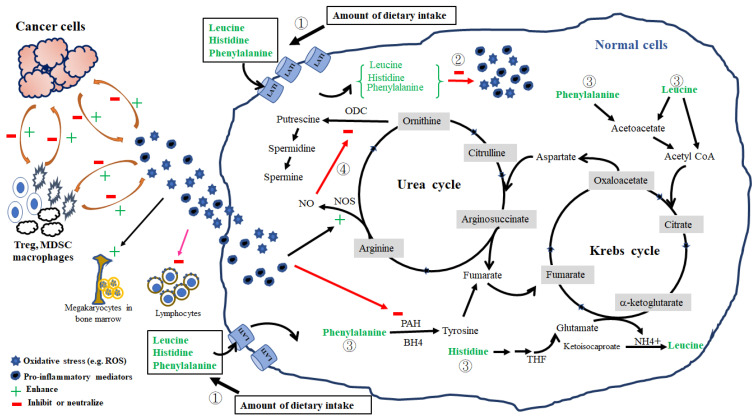
Four possible mechanisms responsible for serum levels of histidine, leucine, ornithine, and phenylalanine in patients with LAHNSCC before concurrent chemoradiotherapy. They include: amount of dietary intake (①), consumption in the neutralization against oxidative and pro-inflammatory stress (②), fuel provider for energy production (③), and nitric oxide synthase (NOS)–nitric oxide (NO) pathway (④). First, reduced dietary intake directly results in decreased serum levels of essential amino acids (histidine, leucine, and phenylalanine). Second, essential amino acids are delivered into cells via L-type amino acid transporter 1 (LAT1); once inside, they can neutralize the reactive oxygen species (ROS) and pro-inflammatory mediators using their antioxidative and anti-inflammatory activities or enter into the Krebs cycle for energy production to compensate calorie deficiency from inadequate food intake. Finally, ROS and pro-inflammatory mediators can stimulate thrombopoiesis from mature megakaryocytes in the bone marrow, inhibit lymphocyte differentiation and proliferation, and upregulate NOS and NO production. Produced NO can decrease ornithine decarboxylase (ODC) activity and increase the ornithine levels. The magnitude of oxidative stress and pro-inflammatory mediators are reciprocally regulated by cancer and immune cells, such as regulatory T cells (Treg), macrophages, and myeloid-derived suppressor cells (MDSC), within the tumor microenvironment and in the peripheral blood. ROS and pro-inflammatory mediators can reduce the function of phenylalanine hydroxylase (PAH) and prevent the conversion of phenylalanine to tyrosine. Tetrahydrobiopterin (BH4) and tetrahydrofolate (THF) are essential cofactors for the conversion of phenylalanine into tyrosine and histidine into glutamate, respectively.

Multivariate analysis further showed the factors affecting the pretreatment levels of individual amino acids in the HLOP panel in patients with cancer. We found that histidine levels were positively associated with levels of phenylalanine but negatively associated with the GPS; leucine levels were positively associated with prealbumin, fasting sugar, and phenylalanine levels but negatively associated with ornithine levels; ornithine levels were positively associated with PLR; phenylalanine levels were positively associated with levels of leucine and ornithine and histologic grade but negatively associated with TLC (Table S1). Based on these observations, the following possible mechanisms, affecting the changes in pretreatment HLOP levels and association with GPS, are proposed. First, HNC-induced dietary intake problems, such as dysphagia and chewing difficulty, result in inadequate ingestion and storage of essential amino acids, which, consequently, affect protein synthesis and turnover. The leucine levels in blood act as a surrogate indicator of the total amount of all essential amino acids [63]. The levels of albumin, prealbumin, Hb, and three essential amino acids (histidine, leucine, and phenylalanine) were found to be reduced in this study, indicating insufficient dietary intake (Figure 1 and Table S1). The positive connection between leucine, phenylalanine, and ornithine in patients with cancer further accentuates the inadequacy and redistribution of leucine and other essential amino acids in mending protein loss, resulting in a decrease in ornithine loads from amino acids. Hence, a lack of essential amino acid supply from dietary intake certainly gave rise to a pretreatment malnutrition status in patients with LAHNSCC. Second, the intimate relationship between histidine, leucine, and phenylalanine can be further strengthened by the fact that they share a common membrane transporter, L-type amino acid transporter 1 (LAT1), which is responsible for the delivery of histidine, phenylalanine, and branched-chain amino acids (BCAAs) into cells and is mainly related to protection against inflammatory stress [64,65]. LAT1 is usually over-expressed in patients with oral cavity squamous cell carcinoma [66]. Through LAT1-mediated delivery into the cells, these three essential amino acids can exert anti-inflammatory effect [67,68,69] or enter the Krebs cycle for energy production to maintain calorie needs from inadequate food intake [70]. Histidine acts as an anti-inflammatory agent by eliminating the NF-κB-mediated production of pro-inflammatory cytokines [69] or suppressing prostaglandin E2 (PGE2) function by interacting with its imidazole ring [71]. CRP production has been shown to be strongly associated with pro-inflammatory cytokines in patients with HNC [72,73]. Histidine is also one of the major amino acids and is involved in the modulation of redox and inflammation of albumin [74]. Thus, the observed negative correlation between histidine and GPS is clear. Leucine, a BCAA, is obtained by diet or from the breakdown of tissue proteins. It acts as the major nitrogen donor to build up the body proteins commonly observed in tissues such as the liver, pancreas, and skeletal muscle [75,76]; leucine reduces oxidative and inflammatory stress in chronic illness [68]. The shift of leucine from blood into the intracellular microenvironment via LAT1 resulted in a decrease in pretreatment serum leucine levels in patients with LAHNSCC, contributing to either the repair of tissue protein damage by oxidative and inflammatory stress from cancer or regulation of energy homeostasis [77]. Since leucine and its metabolites, such as α-ketoisocaproate, are insulin secretagogues [78], an association between fasting sugar and serum leucine levels is possible (Table S1). Additionally, the expression of LAT1 is inversely correlated with the grade of histological differentiation in patients with HNC, which might affect the serum levels of essential amino acids [66]. This implication is supported by the positive association between phenylalanine and histological differentiation observed in our study (Table S1). Although insufficient intake may explain the relationship between phenylalanine, leucine, and ornithine, the negative correlation between phenylalanine and TLC and the positive correlation between ornithine and PLR indirectly reflect the cumulative response of oxidative and inflammatory mediators from cancer itself and/or immune cells in patients with HNC [53,54]. Released from HNC and immune cells, vascular endothelial growth factor (VEGF) directly inhibits T cell development, including thymocyte maturation and differentiation [79], while IL-6 promotes platelet formation [80]. Further, PGE2 released from HNC cells can autocrinally induce the production of hypoxia-inducible factors and VEGF, which, subsequently, generates reactive oxygen species (ROS) from regulatory T cells, macrophages, and myeloid-derived suppressor cells within tumor tissue and in the peripheral blood [81,82]. The balance between the consumption and production of ROS determines T cell proliferation and function [81] and the initiation of thrombopoiesis from mature megakaryocytes in the bone marrow [83]. Similarly, phenylalanine has the antioxidant ability to neutralize ROS-induced oxidative damage via NF-κB-mediated signaling [67], but its intracellular conversion to tyrosine is impaired, probably by ROS-related nitric oxide synthase (NOS) dysregulation and tetrahydrobiopterin depletion [84,85]. Furthermore, pro-inflammatory cytokines and VEGFs synergistically upregulate the NOS activity and induce nitric oxide (NO) production [86,87]. The NO-mediated inhibition of ornithine decarboxylase activity, which is the rate-limiting enzyme of the ornithine degradation pathway and is responsible for the conversion of ornithine to putrescine [88], can increase serum ornithine levels. The orchestrated effect of these mediators, cancer cells, and immune cells on the magnitude of oxidative and inflammatory stress probably determines the serum levels of histidine, leucine, phenylalanine, and ornithine. We have summarized and illustrated the possible mechanisms affecting the serum levels of the pretreatment HLOP panel in Figure 2. Therefore, the HLOP panel provides a summary of circulating amino acids and reveals the nutritional status and inflammation in patients with LAHNSCC before CCRT.

Some intriguing issues merit further investigation. First, the superiority of the GPS and mGPS in prognostic prediction has been compared and debated in several cancer types, including HNC [89,90,91,92,93,94]. However, the results failed to provide a consensus. The major difference between the GPS and mGPS is the risk classification of patients with hypoalbuminemia without an increased CRP levels; these patients are considered an intermediate-risk group in GPS (GPS 1), while they are at low risk in mGPS (mGPS 0). Although mGPS can be a useful prognostic biomarker in patients with HNC in various clinical scenarios [23,25,26,94,95], mGPS classification showed results similar to GPS (Tables S2 and S3), and the systemic inflammation indicated by CRP levels led to escalated protein breakdown and subsequent hypoalbuminemia [96,97,98]. In this study, we still preferred GPS to mGPS due to following reasons. First, patients with HNC developed hypoalbuminemia, probably due to diet intake problems and essential amino acid insufficiency rather than the inflammation. Reduced serum levels of histidine, leucine, and phenylalanine affected albumin, prealbumin, and Hb concentrations in our study, supporting this inference. Although hypoalbuminemia may be partially explained by CRP-mediated systemic inflammation, our data showed that the correlation between albumin and NLR (*p* = 0.023, Pearson correlation) was more significant than that between albumin and CRP (*p* = 0.078, Pearson correlation), and PLR was positively associated with serum ornithine levels rather than CRP levels. Thus, it is plausible that certain inflammatory processes irrelevant to CRP levels may change the serum albumin levels. Hence, the effect of hypoalbuminemia without elevated CRP levels on the risk and prognosis of patients with HNC should be addressed in the analysis. Second, in this study, we found that, in addition to serum histidine levels, age ≥ 65 years was an independent factor associated with pretreatment advanced GPS status (GPS ≥ 1) in patients with LAHSCC (Table 4). Further analysis showed that patients aged ≥ 65 years had significantly lower albumin levels than that of those aged < 65 years (3.44 ± 0.52 vs. 3.92 ± 0.42, respectively; *p* = 0.015), while no difference was found in CRP levels between the two age groups (7.92 ± 0.45 vs. 6.63 ± 8.83, respectively; *p* = 0.733). Gom et al. analyzed more than 60,000 Japanese community-dwelling residents and found a significant decline in serum albumin levels (0.012–0.015 g/dL per year); significantly greater decline was observed in those aged ≥ 65 years: 1.2% and 3.1% in those aged 65–74 and 85–89 years, respectively [99]. The positive association between age ≥ 65 years and pretreatment advanced GPS can be ascribed to hypoalbuminemia. Additionally, serum histidine levels are associated with dietary intake and protein synthesis [100,101]. Our data showed that reduced histidine levels are associated with low albumin levels and advanced GPS but not with the muscle mass. Thus, it is less likely that pretreatment-driven reduction in serum histidine levels is a consequence of an increased shift into muscle, where histidine is converted to carnosine against oxidative stress [102] and subsequently to methylhistidine for muscle synthesis [103]. Finally, decreased serum levels of histidine, leucine, and phenylalanine before treatment may have therapeutic implications. For instance, the histidine degradation process is critical for determining methotrexate efficacy in tumor cells, and addition of histidine to methotrexate significantly hampers the growth of tumors in vivo [104]. Exogenous histidine treatment can reverse sorafenib resistance and enhance anti-tumor activity against hepatocellular carcinoma via LAT1 modulation [105]. In addition, supplementation with certain amino acids aids protein synthesis in patients with cancer cachexia [106]. Although supplements of these essential amino acids before and during the anti-cancer treatment course might avoid treatment failure and mend malnutrition status, this clinical application should be applied carefully as radiation and chemotherapeutic agents generate ROS and inflammation that mainly eradicate cancer cells but also cause toxicity during the treatment course. Whether exogenous amino acids with antioxidative abilities, such as histidine, enhance or comprise the treatment outcomes remains to be investigated.

This study had several limitations. First, this was a single-center study, and, hence, the selection bias should be considered. Although the advantages of this study included the prospective design, homogenous enrollment, standard data collection and treatment protocol, and precise sample size calculation following the head and neck cancer registry of our institute, an advanced, prospectively designed, large-scale, multi-institutional study is required. Second, the study participants, including healthy controls and patients with LAHNSCC, were Taiwanese (predominantly male), had locally advanced cancer status, presented optimal medical performance status, and did not express human papilloma virus. Hence, it is uncertain whether the results of this study can be applied to female patients, non-Taiwanese patients, or patients with varied cancer types, tumor stages, and positive expression of human papilloma virus. Finally, this study lacked a comprehensive profile of the circulating amino acids before CCRT. This drawback may explain the results on the correlation between histidine and GPS and factors associated with the debated HLOP panel. However, even if all amino acid metabolites are produced and the associations are changed, the result regarding the decrease in the levels of certain essential amino acids before CCRT and the association of certain amino acids possessing antioxidative and anti-inflammatory functions with GPS still remains valid.

## 5. Conclusions

This prospective, observational study demonstrated that pretreatment advanced GPS status correlated with low serum histidine levels and 3-year mortality rates in patients with LAHNSCC before CCRT, indicating a close association between inflammation, circulating metabolites, and cancer mortality. Importantly, even with the same tumor stage and medical performance status, patients who already have poor nutrition and high inflammation severity at the beginning of the treatment course may present a more aggressive clinical course and, consequently, show worse survival outcomes than those who have adequate nutrition and low inflammation severity. The assessment of nutritional and inflammatory status, thus, should be routinely performed and considered in future clinical trials to avoid selection bias.

## Figures and Tables

**Table 1:** Clinical features, anthropometric data, biochemical data, nutrition-inflammation biomarkers measurements, and serum metabolites concentrations in the healthy control and patients with LAHNSCC groups.

Variables, Number (%) or Mean ± SD	Control Group	LAHNSCC Group	*p*-Value *
Included participant number	43	50	
Age (years)	53.6 ± 5.9	54.9 ± 8.9	0.516
Male:Female	37 (86.0):6 (14.0)	48 (96.0):2 (4.0)	0.098
Smoking exposure (no:yes)	32 (74.4):11 (25.6)	8 (16.0):42 (84.0)	<0.001 *
Alcohol consumption (no:yes)	32 (74.4):11 (25.6)	14 (28.0):36 (72.0)	<0.001 *
Betel quid use (no:yes)	43 (100.0):0 (0.0)	19 (38.0):31 (62.0)	<0.001 *
Comorbid illness			
Diabetes mellitus (no:yes)	43 (100.0):0 (0.0)	38 (76.0):12 (24.0)	0.001 *
Hypertension (no:yes)	43 (100.0):0 (0.0)	33 (75.0):11 (25.0)	0.001 *
Dyslipidemia (no:yes)	41 (95.4):2 (4.6)	45 (90.0):5 (10.0)	0.366
Congestive heart failure (no:yes)	43 (100.0):0 (0.0)	44 (100.0):0 (0.0)	---
Cardiovascular accident (no:yes)	43 (100.0):0 (0.0)	46 (92.0):4 (8.0)	0.068
Chronic obstructive pulmonary disease (no:yes)	43 (100.0):0 (0.0)	44 (100.0):0 (0.0)	---
Liver cirrhosis with no decompensation (no:yes)	42 (97.7):1 (2.3)	38 (76.0):12 (24.0)	0.003 *
**Anthropometric data**			
BH (m)	1.66 ± 0.05	1.66 ± 0.06	0.829
BW (kg)	69.9 ± 10.8	64.0 ± 11.9	0.272 *
BWL ≤ 5%:>5%	42 (97.7):1 (2.3)	37 (74.0):13 (26.0)	0.001 *
BMI (kg/m^2^)	25.2 ± 3.0	22.9 ± 3.8	0.015 *
<18.5:≥18.5	0 (0.0):43 (100.0)	9 (18.0):41 (82.0)	0.003 *
**Biochemical data**			
eGFR (mL/min/1.73 m^2^)	95.5± 18.8	101.6± 29.8	0.237
ALT (U/L, normal ≤ 36)	29.0± 6.9	22.7± 15.7	0.114
Total bilirubin (mg/dL, normal ≤ 1.3)	0.9± 0.3	0.8± 0.5	0.380
Uric acid (mg/dL, normal < 7.0)	6.0± 1.2	5.2± 1.7	0.008 *
Sugar (fasting, mg/dL)	99.7± 9.5	114.6± 46.5	0.032 *
**Nutrition-Inflammation Biomarkers**			
Hb (g/dL)	14.8 ± 1.3	12.0 ± 1.8	<0.001 *
WBC (×10^3^ cells/mm^3^)	6.5± 1.7	6.6± 2.8	0.930
Platelet count (×10^3^/mm^3^)	222.2 ± 57.2	261.3 ± 88.7	0.013 *
TLC (×10^3^ cells/mm^3^)	2.1± 0.9	1.8± 0.6	0.007 *
<1.5:≥1.5	6 (14.0):37 (86.0)	17 (34.0): 33(66.0)	<0.001 *
TNC (×10^3^ cells/mm^3^)	3.8± 1.3	4.0 ± 2.0	0.462
TMC (×10^3^ cells/mm^3^)	0.3± 0.2	0.5 ± 0.3	0.045 *
Teso (cells/mm^3^)	194.9 ± 24.1	202.5 ± 42.9	0.590
Tbaso (cells/mm^3^)	33.2± 28.1	32.8± 21.9	0.954
Albumin (g/dL, normal:3.5–5.5)	4.4± 0.3	3.9± 0.4	<0.001 *
<3.5:≥3.5	0 (0.0):43 (100.0)	7 (14.0):43 (86.0)	<0.001 *
Total cholesterol (mg/dL, normal < 200)	213.5± 34.9	170.0 ± 42.9	<0.001 *
Triglycerides (mg/dL, normal < 150)	147.2± 91.6	157.2± 97.9	0.629
CRP (mg/L)	1.6± 2.6	6.8± 8.5	<0.001 *
<5:≥5	42 (97.7):1 (2.3)	33 (66.0): 7 (34.0)	0.001^*^
NLR	1.8 ± 0.6	2.9± 4.2	0.075
PLR	108.4± 43.8	166.8± 85.3	<0.001 *
PNI	47.4± 16.9	47.6± 6.1	0.934
GPS 0:1:2	42 (97.7):1 (2.3):0 (0.0)	33 (66.0):13 (26.0):4 (8.0)	0.001 *
**Serum HLOP Metabolites**			
Histidine (μM)	92.8 ± 16.1	78.4 ± 16.2	<0.001 *
Leucine (μM)	152.8 ± 20.2	127.8 ± 38.9	<0.001 *
Ornithine (μM)	93.6 ± 17.1	122.1 ± 34.2	<0.001 *
Phenylalanine (μM)	68.4 ± 12.4	62.9 ± 11.5	0.016 *

* For each variable, *p*-value was determined based on the difference between healthy controls and patients with LAHNSCC; *p* < 0.05, statistical significance. Nonparametric statistics were obtained using Mann–Whitney U test for platelet count, Teso, Tbaso, ALT, uric acid, sugar (fasting), CRP, NLR, PLR, PNI, and all metabolites. Independent *t*-test was implemented for other continuous variables, while chi-square test was used for analyzing sex, smoking, alcohol, betel quid, all comorbid illnesses, BWL (cutoff: 5%), BMI (cutoff: 18.5 kg/m^2^), TLC (cutoff: 1.5 × 10^3^ cells/mm^3^), albumin (cutoff: 3.5 g/dL), CRP (cutoff: 5 mg/L), and GPS. Abbreviations: SD, standard deviation; LAHNSCC, local advanced head and neck squamous cell carcinoma; BH, body height; BW, body weight; BWL, body weight loss; BMI, body mass index; eGFR, estimated glomerular filtration rate; ALT, alanine aminotransferase; Hb, hemoglobin; WBC, white blood cell; TLC, total lymphocyte count; TNC, total neutrophil count; TMC, total monocyte count; Teso, total eosinophil count; Tbaso, total basophil count; CRP, C-reactive protein; NLR, neutrophil-to-lymphocyte ratio; PLR, platelet-to-lymphocyte ratio; PNI, prognostic nutritional index; GPS, Glasgow prognostic score; HLOP, histidine, leucine, ornithine, and phenylalanine.

**Table 2:** Clinicopathologic variables, biochemical and nutrition-inflammation data, anthropometric and body composition characteristics, and serum HLOP metabolite of 50 patients with LAHNSCC stratified by GPS status.

Variables, Numbers (%) or Mean ± SD	ALL	GPS 0	GPS 1	GPS 2	*p*-Value *
Included patient number	50 (100.0)	33 (66.0)	13 (26.0)	4 (8.0)	
**Clinicopathologic**					
Age (years)	54.9 ± 8.9	53.6 ± 8.7	57.3 ±9.1	57.8 ± 10.2	0.367
<65:≥65	44 (88.0):6 (12.0)	32 (97.0):1 (3.0)	9 (69.2):4 (30.8)	3 (75.0):1 (25.0)	0.024 *
Sex (male:female)	48 (96.0):2 (4.0)	32 (97.0):1 (3.0)	12 (92.3):1 (7.7)	4(100.0):0 (0.0)	0.702
Tumor site					0.072
Oral cavity	28 (56.0)	20 (60.6)	7 (53.8)	1 (25.0)	
Oropharynx	9 (18.0)	6 (18.2)	1 (7.7)	2 (50.0)	
Hypopharynx	11 (22.0)	6 (18.2)	5 (3.5)	0 (0.0)	
Larynx	2 (4.0)	1 (3.0)	0 (0.0)	1 (25.0)	
TNM stage					0.671
III IVA IVB	4 (8.0) 23 (46.0) 23 (46.0)	3 (9.1) 17 (51.5) 13 (39.4)	1 (7.7) 5 (38.5) 7 (53.8)	0 (0.0) 1 (25.0) 3 (75.0)	
T status					0.123
T0–2 T3–4	15 (30.0) 35 (70.0)	11 (33.3) 22 (66.7)	2 (15.4) 11 (84.6)	2 (50.0) 2 (50.0)	
N status					0.936
N0–1 N2–3	16 (32.0) 34 (68.0)	11 (33.3) 22 (66.7)	4 (30.8) 9 (69.2)	1 (25.0) 3 (75.0)	
Histological differentiation grade					
Well Moderate Poor	6 (12.0) 37 (74.0) 7 (14.0)	5 (15.2) 23 (69.6) 5 (15.2)	1 (7.7) 11 (84.6) 1 (7.7)	0 (0.0) 3 (75.0) 1 (25.0)	0.739
ECOG performance status					0.024 *
0 1 2	3 (6.0) 43 (86.0) 4 (8.0)	2 (6.1) 30 (90.9) 1 (3.0)	1 (7.7) 11 (84.6) 1 (7.7)	0 (22.2) 2 (50.0) 2 (50.0)	
Tracheostomy					0.159
No Yes	30 (60.0) 20 (40.0)	19 (57.6) 14 (42.8)	10 (76.9) 3 (23.1)	1 (25.0) 3 (75.0)	
Smoking exposure					0.443
No Yes	8 (16.0) 42 (84.0)	6 (18.2) 27 (81.8)	2 (15.4) 11 (84.6)	0 (0.0) 4 (100)	
Alcohol consumption					0.470
No Yes	14 (28.0) 36 (72.0)	11 (33.3) 22 (66.7)	2 (15.4) 11 (84.6)	1 (25.0) 3 (75.0)	
Betel quid use					0.018 *
No Yes	19 (38.0) 31 (62.0)	16 (48.5) 17 (51.5)	3 (23.1) 10 (76.9)	0 (0.0) 4 (100.0)	
HN-CCI					0.233
0 ≥1	20 (40.0) 30 (60.0)	16 (48.5) 17 (51.5)	3 (23.1) 10 (76.9)	1 (25.0) 3 (75.0)	
PG-SGA assessment before CCRT					0.948
Malnutrition none moderate severe	1 (2.0) 21 (42.0) 28 (56.0)	1 (3.0) 14 (42.4) 18 (54.6)	0 (0.0) 5 (38.5) 8 (61.5)	0 (22.2) 2 (50.0) 2 (50.0)	
**Biochemical data**					
eGFR (mL/min/1.73 m^2^)	101.6 ± 29.8	109.8 ± 29.5	97.2 ± 29.4	104.8 ± 14.3	0.408
ALT (U/L, normal ≤ 36)	22.7 ± 15.7	26.5 ± 15.8	20.3 ± 10.6	37.0 ± 23.9	0.153
Total bilirubin (mg/dL, normal ≤ 1.3)	0.8 ± 0.5	0.4 ± 1.7	0.8 ± 0.9	0.4 ± 0.3	0.148
Uric acid (mg/dL, normal < 7.0)	5.2 ± 1.7	5.6 ± 1.7	4.7 ± 1.6	3.7 ± 2.0	0.074
Sugar (fasting, mg/dL)	114.6 ± 46.5	111.8 ± 45.9	125.2 ± 54.1	108.9 ± 21.6	0.606
**Anthropometric and** **blood NIB data**					
BW (kg)	64.0± 11.9	64.3 ±11.3	64.4 ±13.7	60.1 ±13.4	0.755
BWL ≤5% >5%	37 (74.0) 13 (26.0)	24 (72.7) 9 (27.3)	11 (84.6) 2 (15.4)	2 (50.0) 2 (50.0)	0.370
BMI (kg/m^2^)	22.9 ± 3.8	22.9 ±3.8	23.1 ±3.7	22.1 ±4.1	0.924
<18.5 ≥18.5	9 (18.0) 41 (82.0)	6 (18.2) 27 (81.8)	1 (7.7) 12 (92.3)	2 (50.0) 2 (50.0)	0.252
Hb (g/dL)	12.0 ± 1.8	12.3 ±1.5	11.8 ±2.3	9.8 ±1.3	0.027 *^b^
WBC (×10^3^ cells/mm^3^)	6.6± 2.8	6.0 ±1.4	8.0 ±4.4	6.4 ±2.5	0.189
Platelet count (×10^3^/mm^3^)	261.3 ± 88.7	257.5 ± 75.4	293.9 ± 99.3	207.7 ± 108.3	0.197
TLC (×10^3^ cells/mm^3^)	1.8± 0.6	1.9 ±0.5	1.8 ±0.8	1.6± 0.7	0.189
17 (34.0) 33 (66.0)	<1.5 ≥1.5	9 (27.3) 24 (72.7)	5 (38.5) 8 (61.5)	3 (75.0) 1 (25.0)	0.151
TNC (×10^3^ cells/mm^3^)	4.0 ± 2.0	3.5 ±1.1	5.2 ±3.1	4.6 ± 2.0	0.061
TMC (×10^3^ cells/mm^3^)	0.5 ± 0.3	0.4 ±0.1	06 ±0.5	0.4 ± 0.1	0.080
Teso (cells/mm^3^)	202.5 ± 42.9	195.9 ± 116.4	254.8 ± 64.8	212.7 ± 67.2	0.202
Tbaso (cells/mm^3^)	32.8 ± 21.9	34.5 ± 20.3	35.7.9 ± 25.0	29.5 ± 14.5	0.184
Albumin (g/dL)	3.9 ±0.4	3.9 ±0.3	3.9 ±0.6	2.9 ±0.3	<0.001 *^bc^
<3.5 ≥3.5	7 (14.0) 43 (86.0)	0 (0.0) 33 (100.0)	3 (23.1) 10 (76.9)	4 (100.0) 0 (0.0)	<0.001 *
Prealbumin (g/dL, normal: 20–40)	25.1 ±5.6	26.7 ±4.6	23.1 ±6.3	17.3 ±3.5	0.002 *^b^
Transferrin (g/dL normal: 200–360)	203.1 ± 37.9	208.2 ± 36.1	206.5 ± 31.2	149.8 ± 32.8	0.010 *^bc^
Total cholesterol (mg/dL, normal < 200)	170.0± 42.9	175.6 ± 37.7	172.3 ± 51.5	117.0 ± 15.1	0.032 *^bc^
Triglycerides (mg/dL, normal < 150)	157.2± 97.9	157.0 ± 95.0	172.5 ± 71.7	139.0 ± 57.3	0.536
CRP (mg/L)	6.8 ± 8.5	2.3 ± 1.9	13.0 ± 8.5	23.1 ± 11.1	<0.001 *^abc^
NLR	2.9 ± 4.2	2.0± 0.6	3.1± 1.6	9.9± 3.8	0.001 *^bc^
PLR	166.8 ± 85.3	153.3± 78.7	184.5± 73.8	220.2± 93.1	0.230
PNI	47.6 ± 6.1	49.0± 3.8	49.1± 7.0	34.7± 9.7	<0.001 *^bc^
**DXA-related measurements**					
LBM (kg)	44.1 ±5.7	44.3 ±5.4	43.6 ± 6.8	43.7 ± 5.7	0.914
TFM (kg)	17.3 ±7.2	17.4 ±6.9	18.4 ± 7.5	15.3 ± 8.6	0.415
ASM (kg)	18.4 ±4.2	19.1 ±3.2	18.4 ± 3.3	12.5 ± 5.7	0.008 *^bc^
**Serum HLOP metabolites**					
Histidine (μM)	78.4 ± 16.2	83.3 ± 13.8	71.8 ± 16.9	61.8 ± 16.6	0.007 *^b^
Leucine (μM)	127.8 ± 38.9	127.6 ± 34.9	134.8 ± 49.0	119.9 ± 34.5	0.431
Ornithine (μM)	122.1 ± 34.2	120.8 ± 28.8	129.9 ± 46.1	110.2 ± 34.2	0.462
Phenylalanine (μM)	62.9 ± 11.5	61.3 ± 13.6	69.2 ± 22.4	69.2 ± 14.8	0.528
**Three-year mortality rate (%)**	28.0	15.2	46.2	75.0	0.010 *

* For each variable, statistics were determined by comparing the differences among GPS 0, GPS 1, and GPS 2; *p* < 0.05, statistical significance. ^a^ Statistical significance between GPS 0 and GPS 1. ^b^ Statistical significance between GPS 0 and GPS 2. ^c^ Statistical significance between GPS 1 and GPS 2. Nonparametric data for WBC, TNC, TMC, Teso, CRP, NLR, PLR, PNI, phenylalanine, and ASM were analyzed using the Kruskal–Wallis H test, while analysis of variance (ANOVA) with Bonferroni adjustments was implemented for other continuous variables. Chi-square test was used to analyze sex, smoking, alcohol, betel quid, all comorbid illnesses, BWL (cutoff: 5%), BMI (cutoff: 18.5 kg/m^2^), TLC (cutoff: 1.5 × 10^3^ cells/mm^3^), albumin (cutoff: 3.5 g/dL), CRP (cutoff: 5 mg/L), and 3-year mortality rate. Abbreviations: SD, standard deviation; LAHNSCC, local advanced head and neck squamous cell carcinoma; HLOP, histidine, leucine, ornithine, and phenylalanine; GPS, Glasgow prognostic score; TNM stage, tumor–node–metastasis stage; HN-CCI, head and neck—Charlson comorbidity index; ECOG, Eastern Cooperative Oncology Group; PG-SGA, patient-generated subjective global assessment; NIBs, nutrition-inflammation biomarkers; BW, body weight; BMI, body mass index; eGFR, estimated glomerular filtration rate; ALT, alanine aminotransferase; Hb, hemoglobin; WBC, white blood cell; TLC, total lymphocyte count; TNC, total neutrophil count; TMC, total monocyte count; Teso, total eosinophil count; Tbaso, total basophil count; CRP, C-reactive protein; NLR, neutrophil-to-lymphocyte ratio; PLR, platelet-to-lymphocyte ratio; PNI, prognostic nutritional index; DXA, dual-energy X-ray absorptiometry; LBM, lean body mass; TFM, total fat mass; ASM, appendicular skeletal mass.

**Table 3:** Univariate and multivariate logistic regression analysis of prognostic factors associated with 3-year mortality rate of 50 patients with LAHNSCC.

Variables	Univariate Analysis	Multivariate Analysis	
	Odds Ratio (95% CI)	Odds Ratio (95% CI)	*p*-Value
**Clinicopathologic**			
Sex (ref: male)	1.000 (0.989;1.002)		
Age (years)	1.041 (0.965;1.123)		
Age (ref: ≥ 65 years)	0.147 (0.023;0.974)		
Tumor stage (ref: stage III)	1.600 (0.142;18.011)		
T status (ref: T0–2)	1.110 (0.283;4.282)		
N status (ref: N0–1)	2.072 (0.498;8.804)		
Tumor site (ref: non oral cavity)	0.477 (0.136;1.670)		
Histologic differentiation grade (ref: poorly differentiated)	0.846 (0.135;5.317)		
HN-CCI (ref: 0)	2.000 (0.527;7.584)		
ECOG PS (ref: 0)	3.110 (0.521;10.013)		
Smoking (%) (ref: no)	3.138 (0.349;28.180)		
Alcohol (%) (ref: no)	3.000 (0.576;15.614)		
Betel quid (%) (ref: no)	2.754 (0.354;26.523)		
Tracheostomy (ref: no)	1.179 (0.337;4.125)		
PG-SGA (ref: none)	1.235 (0.835;2.449)		
**Biochemical data**			
eGFR (ml/min/1.73 m^2^)	0.977 (0.953;1.002)		
ALT (U/L)	0.976 (0.927;1.026)		
Total bilirubin (mg/dL)	1.392 (0.460;4.215)		
Uric acid (mg/dL)	0.798 (0.546;1.168)		
Sugar (fasting, mg/dL)	1.001 (0.987;1.014)		
**Anthropometric and blood NIB data**			
BW (kg)	0.985 (0.932;1.040)		
BWL (ref: < 5%)	1.944 (0.506;7.473)		
BMI (kg/m^2^)	0.941 (0.791;1.119)		
BMI (ref: > 18.5 kg/m^2^)	1.384 (0.290;6.415)		
Hb (g/dL)	0.659 * (0.437;0.996)		
WBC (×103 cells/mm^3^)	1.224 (0.935;1.603)		
Platelet (×103/mm^3^)	0.997 (0.989;1.004)		
TLC (×103 cells/mm^3^)	0.999 (0.998;1.004)		
TNC (×103 cells/mm^3^)	1.002 * (1.001;1.033)		
TMC (×103 cells/mm^3^)	1.001 (0.999;1.004)		
Teso (cells/mm^3^)	1.001 (0.997;1.005)		
Tbaso (cells/mm^3^)	0.983 (0.954;1.013)		
Albumin (g/dL)	0.314 (0.074;1.342)		
Prealbumin (g/dL)	0.923 (0.825;1.034)		
Transferrin (g/dL)	0.980 * (0.961;0.998)		
Total cholesterol (mg/dL)	0.983 * (0.966;0.997)		
Triglycerides (mg/dL)	0.997 (0.989;1.014)		
CRP (mg/L)	1.102 * (1.018;1.193)		
NLR	2.045 * (1.030;4.059)		
PLR	1.004 (0.957;1.011)		
PNI	0.907 (0.813;1.008)		
GPS (ref: 0)	6.300 * (1.639;24.212)	6.180 * (1.639;24.212)	0.007 *
**DXA-related measurements**			
LBM (kg)	1.012 (0.953;1.134)		
TFM (kg)	0.951 (0.863;1.048)		
ASM (kg)	0.911 (0.785;1.057)		
**Serum HLOP metabolites**			
Histidine (μM)	0.977 (0.939;1.017)		
Leucine (μM)	0.997 (0.980;1.013)		
Ornithine (μM)	0.997 (0.979;1.016)		
Phenylalanine (μM)	1.008 (0.971;1.046)		

* *p* < 0.05 represents statistical significance. Abbreviations: SD, standard deviation; LAHNSCC, local advanced head and neck squamous cell carcinoma; GPS, Glasgow prognostic score; TNM stage, tumor–node–metastasis stage; HN-CCI, head and neck—Charlson comorbidity index; ECOG, Eastern Cooperative Oncology Group; PS, performance status; PG-SGA, patient-generated subjective global assessment; NIBs, nutrition-inflammation biomarkers; BW, body weight; BMI, body mass index; eGFR, estimated glomerular filtration rate; ALT, alanine aminotransferase; Hb, hemoglobin; WBC, white blood cell; TLC, total lymphocyte count; TNC, total neutrophil count; TMC, total monocyte count; Teso, total eosinophil count; Tbaso, total basophil count; CRP, C-reactive protein; NLR, neutrophil-to-lymphocyte ratio; PLR, platelet-to-lymphocyte ratio; PNI, prognostic nutritional index; DXA, dual-energy X-ray absorptiometry; LBM, lean body mass; TFM, total fat mass; ASM, appendicular skeletal mass; HLOP, histidine, leucine, ornithine, and phenylalanine.

**Table 4:** Univariate and multivariate logistic regression analysis of factors associated with GPS ≥ 1 in 50 patients with LAHNSCC.

Variables	Univariate Analysis	Multivariate Analysis	
	Odds Ratio (95% CI)	Odds Ratio (95% CI)	*p*-Value
**Clinicopathologic**			
Sex (ref: male)	2.000 (0.117;34.092)		
Age (years)	1.055 (0.980;1.136)		
Age (ref: ≥ 65 years)	0.075 * (0.008;0.710)	0.041 * (0.003;0.546)	0.016 *
Tumor stage (ref: stage III)	2.308 (0.208;12.234)		
T status (ref: T0–2)	1.625 (0.428;6.169)		
N status (ref: N0–1)	1.200 (0.337;4.272)		
Tumor site (ref: non oral cavity)	0.578 (0.177;1.882)		
Histologic differentiation grade (ref: poorly differentiated)	0.500 (0.034;1.452)		
HN-CCI (ref: 0)	3.059 (0.824;11.324)		
ECOG PS (ref: 0)	6.000 (0.221;10.423)		
Smoking (%) (ref: no)	1.667 (0.298;6.310)		
Alcohol (%) (ref: no)	2.333 (0.552;9.866)		
Betel quid (%) (ref: no)	1.375* (1.049;3.624)		
Tracheostomy (ref: no)	1.351 (0.403;4.534)		
PG-SGA (ref: none)	4.000 (0.437;36.576)		
**Biochemical data**			
eGFR (ml/min/1.73 m^2^)	0.986 (0.964;1.008)		
ALT (U/L)	0.990 (0.950;1.031)		
Total bilirubin (mg/dL)	8.369 * (1.013;69.919)		
Uric acid (mg/dL)	0.665 * (0.445;0.995)		
Sugar (fasting, mg/dL)	1.004 (0.991;1.016)		
**Anthropometric and blood NIB data**			
BW (kg)	0.993 (0.944;1.045)		
BMI (kg/m^2^)	0.998 (0.850;1.172)		
Hb (g/dL)	0.710 (0.489;1.030)		
WBC (×103 cells/mm^3^)	1.314 (0.956;1.805)		
Platelet (×103/mm^3^)	1.001 (0.995;1.008)		
TLC (×103 cells/mm^3^)	0.999 (0.998;1.002)		
TNC (×103 cells/mm^3^)	1.030 * (1.001;1.200)		
TMC (×103 cells/mm^3^)	1.002 (0.998;1.002)		
Teso (cells/mm^3^)	1.002 (0.996;1.006)		
Tbaso (cells/mm^3^)	0.989 (0.962;1.017)		
Albumin (g/dL)	0.198 * (0.042;0.920)		
Prealbumin (g/dL)	0.835 * (0.732;0.952)		
Transferrin (g/dL)	0.989 (0.972;1.005)		
Total cholesterol (mg/dL)	0.991 (0.976;1.005)		
Triglycerides (mg/dL)	1.000 (0.994;1.006)		
CRP (mg/L)	1.693 * (1.230;2.331)		
NLR	3.655 * (1.437;9.298)		
PLR	1.005 (0.998;1.013)		
PNI	0.890 * (0.798;0.992)		
**DXA-related measurements**			
LBM (kg)	0.977 (0.880;1.085)		
TFM (kg)	0.998 (0.917;1.086)		
ASM (kg)	0.881 (0.751;1.033)		
**Serum HLOP metabolites**			
Histidine (μM)	0.938 * (0.896;0.983)	0.906 * (0.835;0.984)	0.019 *
Leucine (μM)	1.000 (0.985;1.016)		
Ornithine (μM)	1.003 (0.986;1.020)		
Phenylalanine (μM)	1.030 (0.993;1.068)		

* *p* < 0.05 represents statistical significance. Abbreviations: SD, standard deviation; LAHNSCC, local advanced head and neck squamous cell carcinoma; GPS, Glasgow prognostic score; TNM stage, tumor–node–metastasis stage; HN-CCI, head and neck—Charlson comorbidity index; ECOG, Eastern Cooperative Oncology Group; PS, performance status; PG-SGA, patient-generated subjective global assessment; NIBs, nutrition-inflammation biomarkers; BW, body weight; BMI, body mass index; eGFR, estimated glomerular filtration rate; ALT, alanine aminotransferase; Hb, hemoglobin; WBC, white blood cell; TLC, total lymphocyte count; TNC, total neutrophil count; TMC, total monocyte count; Teso, total eosinophil count; Tbaso, total basophil count; CRP, C-reactive protein; NLR, neutrophil-to-lymphocyte ratio; PLR, platelet-to-lymphocyte ratio; PNI, prognostic nutritional index; DXA, dual-energy X-ray absorptiometry; LBM, lean body mass; TFM, total fat mass; ASM, appendicular skeletal mass; HLOP, histidine, leucine, ornithine, and phenylalanine.

## Data Availability

The data presented in this study are available on request from the corresponding author.

## Supplementary Materials

The following supporting information can be downloaded at: https://www.mdpi.com/article/10.3390/nu14173475/s1, Table S1. Associations between clinicopathologic variables, NIBs, and body composition parameters and pretreatment levels of histidine, leucine, ornithine, and phenylalanine in 50 patients with LAHNSCC. Table S2. The mGPS was the only prognostic factor associated with 3-year mortality in 50 patients with LAHNSCC. Table S3. Univariate and multivariate logistic regression analyses of factors associated with an mGPS ≥ 1 in 50 patients with LAHNSCC.
