# Supplementary material for: Pretreatment Glasgow Prognostic Score Correlated with Serum Histidine Level and Three-Year Mortality of Patients with Locally Advanced Head and Neck Squamous Cell Carcinoma and Optimal Performance Status
Source: Nutrients. 2022 Aug 24;14(17):3475. doi: 10.3390/nu14173475 (PMC9458049; doi:10.3390/nu14173475)
Supplement: Supplementary file 1 [file nutrients-14-03475-s001.zip › nutrients-1878070-supplementary.pdf]

Table S1. The associations of clinicopathologic variables, NIBs, and body composition parameters with pretreatment levels of histidine, leucine ornithine, and phenylalanine in 50 patients with LAHNSCC

Variables	Histidine			Leucine			Ornithine			Phenylalanine		
	Univariate	Multivariate		Univariate	Multivariate		Univariate	Multivariate		Univariate	Multivariate	
	p value[1]	Coefficient (95% CI)	*p* value[1]	*p* value[1]	Coefficient (95% CI)	*p* value[1]	*p* value[1]	Coefficient (95% CI)	*p* value[1]	*p* value[1]	Coefficient (95% CI)	*p* value[1]
Clinicopathologic factors												
Age	0.262			0.682			0.0081			0.282		
Sex (male *vs.* female)	0.476			0.641			1.000			0.279		
Tumor location (OC *vs.* NOC)	0.530			0.749			0.349			0.303		
TNM stage (III *vs.* IVA *vs.* IVB)	0.802			0.565			0.260			0.914		
T status (T1-2 *vs.* T3-4)	0.105			0.041[1]			0.088			0.044[1]		
N status (N0-1 *vs.* N2-3)	0.665			0.853			0.757			0.833		
Histologic grade (1 *vs.* 2 *vs.* 3)	0.191			0.842			0.786			0.023[1]	11.208 (2.697 – 19.449)	0.009[1]
Smoking (no *vs.* yes)	0.509			0.683			0.875			0.092		
Alcohol (no *vs.* yes)	0.106			0.043[1]			0.315			0.805		
Betel nut (no *vs.* yes)	0.393			0.826			0.393			0.751		
ECOG performance status (0 : 1 : 2)	0.038[1]			0.072			0.197			0.070		
Tracheostomy (no *vs.* yes)	0.242			0.273			0.419			0.132		
PG-SGA before CCRT (none : moderate : severe)	0.333			0.910			0.085			0.325		
Biochemical data												
eGFR (ml/min/1.73m^2)	0.574			0.554			0.186			0.196		
ALT (U/L)	0.611			0.117			0.154			0.056		
Total bilirubin (mg/dL)	0.824			0.303			0.288			0.145		
Uric acid (mg/dL)	0.009[1]			0.893			0.539			0.224		
Sugar (fasting, mg/dL)	0.963			0.024[1]	0.365 (0.206 – 0.523)	<0.001[1]	0.286			0.054		
Anthropometric data and blood NIBs data												
BW (kg)	0.627			0.676			0.923			0.977		
BMI (kg/m^2)	0.863			0.919			0.901			0.936		
Hb (g/dL)	0.010[1]			0.012*			0.567			0.669		
WBC (x10^{3}cells/mm^3)	0.137			0.922			0.193			0.167		
Platelet count (x10^3/mm^3)	0.511			0.987			0.227			0.259		
TLC (x10^{3}cells/mm^3)	0.879			0.915			0.034[1]			0.030[1]	-0.006 (-0.011 – -0.001)	0.013[1]

TNC (x 10^3/mm^3)	0.108			0.789			0.371			0.373		
TMC (x 10^3/mm^3)	0.116			0.565			0.485			0.390		
Teso (cells/mm^3)	0.895			0.754			0.443			0.855		
Tbaso (cells/mm^3)	0.082			0.095			0.065			0.003[1]		
Albumin (g/dL)	0.038[1]			0.049[1]			0.962			0.797		
Prealbumin (g/dL)	0.060			0.013[1]	3.166 (1.936 – 4.396)	<0.001[1]	0.574			0.484		
Transferrin (g/dL)	0.165			0.266			0.582			0.278		
Total cholesterol (mg/dL)	0.211			0.099			0.671			0.462		
Triglyceride (mg/dL)	0.944			0.127			0.459			0.888		
CRP (mg/L)	0.001[1]			0.999			0.829			0.946		
NLR	0.197			0.747			0.167			0.260		
PLR	0.711			0.974			0.035[1]	0.041 (0.029 – 0.194)	0.009[1]	0.100		
PNI	0.151			0.109			0.512			0.160		
GPS	0.003[1]	-15.232 (-23.091 – -0.751)	<0.001[1]	0.968			0.730			0.107		
DXA-related measurements												
LBM (kg)	0.450			0.912			0.630			0.536		
TFM (kg)	0.675			0.659			0.697			0.725		
ASM (kg)	0.114			0.723			0.709			0.602		
Metabolites												
Before CCRT												
Histidine (μM)	---			0.002[1]			0.002[1]			0.002[1]		
Leucine (μM)	0.002[1]			---			0.013[1]			<0.001[1]	0.203 (0.123 – 0.284)	<0.001[1]
Ornithine (μM)	0.002[1]			0.013[1]	-0.385 (-0.624 – -0.147)	0.002[1]	---			<0.001[1]	0.168 (0.067 – 0.265)	0.001
Phenylalanine (μM)	0.002[1]	0.538 (0.325 – 0.571)	<0.001[1]	<0.001[1]	1.663 (1.164 – 2.163)	<0.001[1]	<0.001[1]			---		

[1]indicates a significant p value of < 0.05.

Univariate analysis: simple linear regression model for all continuous variables; Mann−Whitney test for sex, T status, N status, smoking, alcohol, betel nut, tracheostomy, and feeding tube placement; Kruskal−Wallis test for TNM stage, histological grade, ECOG performance status, and PG-SGA.

‡Δ indicates the value obtained by subtracting the pretreatment value from the post-treatment value. % indicates (Δ value/ the pretreatment value) x 100%.

Abbreviations: NIBs, nutritional and inflammatory biomarkers; LAHNSCC, locally advanced head and neck squamous cell carcinoma; CCRT, concurrent chemoradiotherapy; CI, confidence interval; OC, oral cavity; NOC, non-oral cavity; TNM, tumor node metastasis; ECOG, Eastern Collaboration Oncology Group; RT, radiotherapy; PG-SGA, patient-generated subjective global assessment; eGFR, estimated glomerular filtration rate; ALT, alanine transaminase; NIBs, nutritional/inflammatory biomarkers; BMI, body mass index; BWL, body weight loss; Hb, hemoglobin; WBC, white cell count; TLC, total lymphocyte count; TNC, total neutrophil count; TMC, total monocyte count; CRP, C-reactive protein.

Table S2. mGPS is the only one prognostic factor associated with 3-year mortality in 50 patients with LAHNSCC

	**Univariate analysis**		**Multivariate analysis**	
**Variables**	**Odds ratio (95% CI)**	***p* value[1]**	**Odds ratio (95% CI)**	***p* value[1]**
**Clinicopathologic**				
**Sex (ref: male)**	0.997 (0.889-1.002)	0.849		
**Age (years)**	1.041 (0.965-1.123)	0.300		
**Age (ref: ≥ 65 years)**	0.147 (0.023-0.924)	0.041[1]		
**Tumor stage (ref: stage III)**	1.600 (0.142-18.000)	0.704		
**T status (ref: T0-2)**	1.110 (0.283-4.282)	0.891		
**N status (ref: N0-1)**	2.072 (0.488-8.804)	0.323		
**Tumor site (ref: non oral cavity)**	0.477 (0.136-1.670)	0.247		
**Histologic differentiation grade (ref: poorly differentiated)**	0.846 (0.135-5.317)	0.859		
**HN-CCI (ref: 0)**	2.000 (0.527-7.584)	0.308		
**ECOG PS (ref: 0)**	4.846 (0.111-31.531)	0.399		
**Smoking (%) (ref: no)**	3.138 (0.349–28.180)	0.307		

**Alcohol (%) (ref: no)**	3.000 (0.576-15.614)	0.192
**Betel quid (%) (ref: no)**	1.754 (0.514-26.523)	0.659
**Tracheostomy (ref: no)**	1.179 (0.337-4.125)	0.797
**PG-SGA (ref: none)**	3.4295 (0.372-31.589)	0.461
Biochemical data		
**eGFR (ml/min/1.73m^2)**	0.977 (0.943-1.001)	0.076
**ALT (U/L)**	0.976 (0.927-1.026)	0.340
**Total bilirubin (mg/dL)**	1.392 (0.460-4.215)	0.535
**Uric acid (mg/dL)**	0.798 (0.546-1.168)	0.246
**Sugar (fasting, mg/dL)**	1.001 (0.987-1.014)	0.937
Anthropometric and blood NIB data		
**BW (kg)**	0.985 (0.932-1.040)	0.985
**BWL (ref: < 5%)**	1.944 (0.506-7.473)	0.333
**BMI (kg/m^2)**	0.941 (0.791-1.119)	0.490
**BMI (ref: > 18.5 kg/m^2)**	1.364 (0.290-6.415)	0.695
**Hb (g/dL)**	0.659 (0.437-0.996)	0.048[1]
**WBC (x 10^3 cells/mm^3)**	1.224 (0.935-1.603)	0.142
**Platelet (x 10^3/mm^3)**	0.997 (0.989-1.004)	0.369
**TLC (x 10^3 cells/mm^3)**	0.999 (0.998-1.001)	0.186
**TNC (x 10^3 cells/mm^3)**	1.011 (1.003-1.015)	0.046[1]
**TMC (x 10^3 cells/mm^3)**	1.001 (0.998-1.004)	0.293
**Teso (cells/mm^3)**	1.001 (0.997-1.005)	0.629
**Tbaso (cells/mm^3)**	0.983 (0.954-1.013)	0.258

**Albumin (g/dL)**	0.314 (0.074-1.342)	0.118		
**Prealbumin (g/dL)**	0.923 (0.825-1.034)	0.166		
**Transferrin (g/dL)**	0.980 (0.961-0.999)	0.042[1]		
**Total cholesterol (mg/dL)**	0.983 (0.966-0.998)	0.046[1]		
**Triglyceride (mg/dL)**	0.997 (0.989-1.004)	0.403		
**CRP (mg/L)**	1.102 (1.018-1.193)	0.016[1]		
**NLR**	2.045 (1.030-4.059)	0.041[1]		
**PLR**	1.004 (0.997-1.011)	0.324		
**PNI**	0.907 (0.816-1.008)	0.069		
**mGPS (ref: 0)**	8.267 (2.001-34.155)	0.004[1]	8.776 (1.910-40.329)	0.005[1]
DXA-related measurements				
**LBM (kg)**	1.012 (0.903-1.134)	0.839		
**TFM (kg)**	0.951 (0.863-1.048)	0.307		
**ASM (kg)**	0.911 (0.785-1.057)	0.218		
Serum HLOP Metabolites				
**Histidine (μM)**	0.977 (0.939-1.017)	0.264		
**Leucine (μM)**	0.997 (0.980-1.013)	0.694		
**Ornithine (μM)**	0.997 (0.979-1.016)	0.786		
**Phenylalanine (μM)**	1.008 (0.971-1.046)	0.670		

[1] $p < 0.05$ represents statistical significance.

Abbreviations: SD, standard deviation; LAHNSCC, local advanced head and neck squamous cell carcinoma; mGPS, modified Glasgow prognostic score; TNM stage, tumor-node-metastasis stage; HN-CCI, Charlson Comorbidity Index; ECOG, Eastern Cooperative Oncology Group; PS, performance status; PG-SGA, patient generated subjective global assessment; NIBs, nutrition-inflammation biomarkers; BW, body weight; BMI, body mass index; eGFR, estimated glomerular filtration rate; ALT, alanine aminotransferase; Hb, hemoglobin; WBC, white blood cell; TLC, total lymphocyte count; TNC, total neutrophil count; TMC, total monocyte count; Teso, total eosinophil count; Tbaso, total basophil count; CRP, C-reactive protein; NLR, neutrophil-to-lymphocyte ratio; PLR, platelet-to-lymphocyte ratio; PNI, prognostic nutritional index;

DXA, dual-energy X-ray absorptiometry; LBM, lean body mass; TFM, total fat mass; ASM, appendicular skeletal mass; HLOP, histidine, leucine, ornithine and phenylalanine.

Table S3. Univariate and multivariate logistic regression analysis of factors associated with mGPS ≥ 1 in 50 patients with LAHNSCC

	Univariate analysis		Multivariate analysis	
**Variables**	**Odds ratio (95% CI)**	***p* value[1]**	**Odds ratio (95% CI)**	***p* value[1]**
**Clinicopathologic**				
**Sex (ref: male)**	3.000 (0.174-51.747)	0.632		
**Age (years)**	1.024 (0.950-1.106)	0.350		
**Age (ref: ≥ 65 years)**	0.034 (0.028-0.510)	0.019[1]	0.057(0.008-0.666)	0.012[1]
**Tumor stage (ref: stage III)**	1.600 (0.142-18.000)	0.704		
**T status (ref: T0-2)**	1.600 (0.371-6.906)	0.529		
**N status (ref: N0-1)**	1.080 (0.276-4.025)	0.912		
**Tumor site (ref: non oral cavity)**	0.584 (0.164-2.087)	0.408		
**Histologic differentiation grade (ref: poorly differentiated)**	0.833 (0.041-1.452)	0.585		
**HN-CCI (ref: 0)**	1.714 (0.446-6.583)	0.432		
**ECOG PS (ref: 0)**	4.895 (0.989-41.464)	0.166		
**Smoking (%) (ref: no)**	1.065 (0.186-6.076)	0.944		
**Alcohol (%) (ref: no)**	2.640 (0.504-13.825)	0.251		
**Betel quid (%) (ref: no)**	1.264 (1.071-2.991)	0.048[1]		
**Tracheostomy (ref: no)**	1.917 (0.857-3.350)	0.895		
**PG-SGA (ref: none)**	1.938 (0.371-19.318)	0.912		
Biochemical data				

**eGFR (ml/min/1.73m^2)**	0.981 (0.956-1.006)	0.126		
**ALT (U/L)**	0.992 (0.949-1.036)	0.992		
**Total bilirubin (mg/dL)**	1.062 (0.317-1.042)	0.922		
**Uric acid (mg/dL)**	0.675 (0.437-1.042)	0.076		
**Sugar (fasting, mg/dL)**	1.007 (0.914-1.020)	0.280		
Anthropometric data and blood NIB data				
**BW (kg)**	0.996 (0.942-1.052)	0.879		
**BMI (kg/m2)**	0.993 (0.944-1.189)	0.921		
**Hb (g/dL)**	0.855 (0.594-1.230)	0.398		
**WBC (x 10^3 cells/mm^3)**	1.506 (1.027-2.208)	0.036[1]		
**Platelet (x 10^3/mm^3)**	1.003 (0.996-1.010)	0.379		
**TLC (x 10^3 cells/mm^3)**	1.000 (0.999-1.001)	0.832		
**TNC (x 10^3 cells/mm^3)**	1.002 (1.001-1.005)	0.010[1]	1.003 (1.001-1.005)	0.029[1]
**TMC (x 10^3 cells/mm^3)**	1.002 (0.999-1.004)	0.246		
**Teso (cells/mm^3)**	1.002 (0.998-1.006)	0.293		
**Tbaso (cells/mm^3)**	0.987 (0.957–1.017)	0.386		
**Albumin (g/dL)**	0.133 (0.032-0.950)	0.041[1]		
**Prealbumin (g/dL)**	0.914 (0.814-1.027)	0.131		
**Transferrin (g/dL)**	0.990 (0.972-1.008)	0.258		
**Total cholesterol (mg/dL)**	0.996 (0.989-1.011)	0.563		
**Triglyceride (mg/dL)**	1.001 (0.995-1.080)	0.689		
**CRP (mg/L)**	1.023 (1.011-2.450)	0.001[1]		
**NLR**	1.785 (0.956-3.331)	0.069		

**PLR**	1.003 (0.996-1.011)	0.357		
**PNI**	0.950 (0.818-1.051)	0.318		
DXA-related measurements				
**LBM (kg)**	0.972 (0.869-1.087)	0.617		
**TFM (kg)**	1.007 (0.919-1.103)	0.884		
**ASM (kg)**	0.852 (0.717-1.012)	0.068		
Serum HLOP Metabolites				
**Histidine (μM)**	0.929 (0.881-0.979)	0.006[1]	0.928 (0.875-0.983)	0.012[1]
**Leucine (μM)**	1.008 (0.992-1.024)	0.355		
**Ornithine (μM)**	0.933 (0.973-1.013)	0.495		
**Phenylalanine (μM)**	1.016 (0.979-1.055)	0.495		

[1]$p < 0.05$ represents statistical significance.

Abbreviations: SD, standard deviation; LAHNSCC, local advanced head and neck squamous cell carcinoma; mGPS, modified Glasgow prognostic score; TNM stage, tumor-node-metastasis stage; HN-CCI, Charlson Comorbidity Index; ECOG, Eastern Cooperative Oncology Group; PS, performance status; PG-SGA, patient generated subjective global assessment; NIBs, nutrition-inflammation biomarkers; BW, body weight; BMI, body mass index; eGFR, estimated glomerular filtration rate; ALT, alanine aminotransferase; Hb, hemoglobin; WBC, white blood cell; TLC, total lymphocyte count; TNC, total neutrophil count; TMC, total monocyte count; Teso, total eosinophil count; Tbaso, total basophil count; CRP, C-reactive protein; NLR, neutrophil-to-lymphocyte ratio; PLR, platelet-to-lymphocyte ratio; PNI, prognostic nutritional index; DXA, dual-energy X-ray absorptiometry; LBM, lean body mass; TFM, total fat mass; ASM, appendicular skeletal mass; HLOP, histidine, leucine, ornithine and phenylalanine.
